# Assessing the Potential for Integrating Routine Data Collection on Complementary Feeding to Child Health Visits: A Mixed-Methods Study

**DOI:** 10.3390/ijerph16101722

**Published:** 2019-05-16

**Authors:** Louise Tully, Charlotte M. Wright, Deirdre McCormick, Ada L. Garcia

**Affiliations:** 1 Human Nutrition, School of Medicine, Dentistry and Nursing, College of Medical, Veterinary and Life Sciences, University of Glasgow, Glasgow G31 2ER, UK; LMTully1@gmail.com; 2 Child Health, School of Medicine, Dentistry and Nursing, College of Medical, Veterinary and Life Sciences, University of Glasgow, Glasgow G51 4TF, UK; charlotte.wright@glasgow.ac.uk; 3 NHS Greater Glasgow and Clyde, East Renfrewshire and Inverclyde Health and Social Care Partnerships, Glasgow G31 2ER, UK; Deirdre.McCormick@ggc.scot.nhs.uk

**Keywords:** infant nutrition, complementary feeding, diet, early years, public health nutrition interventions, feeding behavior

## Abstract

There is no routine data collection in the UK on infant dietary diversity during the transition to solid foods, and health visitors (HVs) (nurses or midwives with specialist training in children and family health) have the potential to play a key role in nutrition surveillance. We aimed to assess items for inclusion in routine data collection, their suitability for collecting informative data, and acceptability among HVs. A mixed-methods study was undertaken using: (i) an online survey testing potential questionnaire items among parents/caregivers, (ii) questionnaire redevelopment in collaboration with community staff, and (iii) a survey pilot by HVs followed by qualitative data collection. Preliminary online questionnaires (*n* = 122) were collected to identify useful items on dietary diversity. Items on repeated exposure to foods, aversive feeding behaviors, flavor categories, and sugar intake were selected to correspond to nutrition recommendations, and be compatible with electronic records via tablet. HVs surveyed 187 parents of infants aged 12 months. Semi-structured interviews indicated that HVs found the questionnaire comparable with standard nutrition conversations, which prompted helpful discussions, but questions on eating behavior did not prompt such useful discussions and, in some cases, caused confusion about what was ‘normal.’ Lack of time among HVs, internet connectivity issues, and fear of losing rapport with parents were barriers to completing electronic questionnaires, with 91% submitted by paper. Routine nutrition data collection via child health records seems feasible and could inform quality improvement projects.

## 1. Introduction

The transition to solid feeding is an important aspect of development during infancy. Recommendations advise exclusive breastfeeding for the first six months of life, which is followed by increased nutrient density by introducing complementary solid foods [1,2]. During the complementary feeding period, increasing the variety of foods and textures is important for healthy growth and to set the foundations for food preferences and healthy eating later in life [3,4]. It has been documented in the literature that parents/caregivers find the complementary feeding period challenging to navigate [5,6]. Health visitors have been identified for their key role in providing infant feeding advice as relied upon by parents/caregivers [7,8] and for their potential to encourage appropriate feeding for preventing obesity [9]. A health visitor (HV) is a qualified nurse (or midwife) who has completed specialist training in children and family health. HVs offer support and advice regarding the wellbeing of children until the school years. HVs routinely discuss infant feeding with parents/caregivers as part of the universal Health Visiting Pathway in Scotland from pre-birth to preschool [10]. The program consists of eleven home visits to be delivered by HVs to all families, eight of which are within the first year of life, and three Child Health Reviews between 13 months and 4–5 years. However, these infant nutrition conversations are not systematically delivered or recorded, and could provide an opportunity for the collection of routine data related to nutrition and dietary diversity. Moreover, health workers’ dietary counselling has been shown to have a potential positive influence on infant feeding practices [11], and establishing routine, consistent conversations around feeding practices could provide an opportunity for intervention and/or evaluations of earlier interventions.

Within the majority of National Health Service (NHS) board areas across Scotland, HV teams now use electronic patient recording systems via tablets and computers. EMIS Web is the key cornerstone Electronic Patient Record Application in Community Services in NHS Greater Glasgow and Clyde (GGC) providing a single shared record for community-based children’s services. During this research study, EMIS was in the process of being rolled out to all Children and Family teams. Electronic record keeping is advocated to improve administrative efficiency and time management [12,13,14], and this provides a suitable opportunity to incorporate consistent and valid data collection. Nutrition surveillance at a population level allows practitioners to gather evidence to inform policy and practice [15], and recent surveys such as the Scottish maternal and infant nutrition survey [16] and the previous five-yearly UK infant feeding survey [17] demonstrate this. Widespread routine collection of similar information would allow for the maintenance of nationally representative and up-to-date nutrition data on demand.

The present study, thus, aimed to explore the feasibility of developing a group of questions about dietary intake and behavior during the complementary feeding period. We set out to develop a tool, which reflected both the World Health Organization’s indicators for assessing infant and young child feeding practices [18] and specific UK recommendations [19], while having the potential to be recorded in electronic health records. We also aimed to explore their utility in a routine field setting, using an iterative process to identify the most appropriate survey items.

## 2. Materials and Methods

This was a mixed methods study, consisting of an explanatory sequential design, of which there were three stages (Table 1).

### 2.1. Online Survey

#### 2.1.1. Recruitment & Data Collection

We recruited a convenience sample online, using websites and social media pages aimed at parents and caregivers of young children between 9–15 months in Scotland. We contacted all site/page administrators initially where necessary, and obtained permission to post a link to the questionnaire. The research team set up an email address and Facebook page specifically for the study to provide information for parents. All online recruitment posts directed potential participants to the public study Facebook page, with an online participant information sheet.

A questionnaire was compiled using items drawn from the Gateshead Millennium Cohort Study [20,21] (supplementary material A) and converted it into an online version using the ‘Qualtrics’ [22] online survey platform.

The questionnaire required electronic acknowledgment of informed consent, and participants were made aware that all information they submitted would be kept anonymous. The questions assessed the type of foods offered to infants (frequency and type of beverages and fruit/vegetables, which fruit/vegetables they have tried, and frequency of meat, fish, and eggs). They also assessed the feeding methods used (self-fed finger foods vs. spoon-fed), as well as infant responses (frequency of crying, turning, spitting, gagging, pushing food away, holding food in the mouth, or throwing food during meals). We excluded partial or incomplete responses and participants whose children were not between 9 and 15 months old. 

Researchers obtained ethical approval (application No. 200140137) from the University of Glasgow, College of Medical, Veterinary and Life Sciences research ethics committee for the online survey.

#### 2.1.2. Data Analysis

Since a much shorter tool was needed for incorporation into the HV workload, we used descriptive statistics to give an overview of the infant dietary diversity and the frequency of different feeding styles/infant responses, in order to identify the most useful and discriminating questions. We did not have enough data to perform a formal factor analysis for combining survey items, and, therefore, we used Spearman’s correlation to assess the extent to which avoidant infant responses to feeding were most closely associated in order to create fewer more focused questions.

### 2.2. Stakeholder Consultation

The research team subsequently consulted key health visiting managers in the NHS GGC board area to discuss the use of the online survey results in redrafting the questionnaire and piloting this with a sample of HVs in the NHS GGC area. The researchers then met with the leads of each HV team to be involved in testing the tool to assess their willingness to take part and take into account their suggestions. All HVs on eligible teams were then given information on the research protocol and the opportunity to address answered queries. Feedback was received on all aspects of the survey and its format, which was incorporated to the redrafted questionnaire (Table 2) before the survey. A nutrition fact sheet (supplementary material B) to accompany the questionnaire was also developed for use by HV teams to guide them when nutritional advice was needed.

### 2.3. Health Visiting Survey

The mobile version of the ‘Questback’ online survey platform [23] was used, in order to replicate as closely as possible the use of the questionnaire in EMIS web, which is accessed via iPad. However, paper questionnaires were offered as alternative, for use when staff members could not access the online questionnaire for any reason. Members of HV teams (including staff nurses, nursery nurses, and Healthcare Support Workers) carried out the survey as a service evaluation among consenting participants either during the routine 12-month visit, or by phone call. 

After the survey, all members of participating HV teams (*n* = 91) were invited by email to take part in a short, semi-structured telephone interview to reflect on their experience of testing the questionnaire in practice. All HV team members who expressed interest were interviewed. The researcher took detailed notes during interviews in order to record participant responses. One additional HV staff member provided feedback via email, which was also included in the analysis. These were later analyzed inductively using a thematic approach [24]. The researcher read and reread the notes taken during interviews and became familiar with the data, before generating initial codes using NVivo 11. During a second round of coding, overlapping and common subject areas were further identified and categorized until overarching themes representing the experiences of HVs were developed.

## 3. Results

### 3.1. Online Survey

Parents/caregivers of 122 infants completed the full online questionnaire. The mean child age was 9.5 months. Eighty-seven (72%) participants cited members of HV teams as sources of ‘some’ or ‘a lot’ of their complementary feeding advice. Infant dietary diversity as reported by parents was generally in line with recommendations, and, in particular, parents reported a high intake of fruit and vegetables with around 90% of infants having each at least once per day. However, the list of possible fruit and vegetables revealed sweet vegetables and the less sour fruit were most popular (Table 3). Furthermore, it became apparent that recording ever-tried fruit and vegetables without frequencies was not informative without some insight to frequency of exposure. To overcome this, and also to continue reducing the number of survey items in line with the study aims, the research team subsequently used this information to group fruit and vegetables into flavor categories in order to asses flavor variety in the infant diet for the HV survey. The categories were grouped as follows: (i) sweet, starchy vegetables, (ii) sour fruit, and (iii) green bitter vegetables, to later be assessed in terms of frequency of exposure. In addition, the open-ended questions in the initial online survey allowed us to ensure we captured all types of fruit and vegetables commonly consumed, which allows us to rephrase our numerical questions for the second survey.

Most of the eight possible avoidant eating behaviors were uncommon and all tended to be inter-correlated (see Table 4). Pushing food away, closing mouth when offered food, and turning head when offered food were strongly inter-correlated (*p* < 0.001). Holding food in mouth, spitting food, and throwing food were also quite strongly correlated. Crying/screaming and gagging were the rarest behaviors and are likely to indicate more serious feeding issues. We, thus, formed four new variables for the HV survey using this grouping: frequency of (i) pushing food away/closing mouth/turning head, (ii) holding food in mouth/spitting food/throwing food, (iii) crying/screaming during feeding, and (iv) gagging on food.

The results for questions related to the frequency of self-feeding vs. spoon-feeding highlighted that long, Likert-scale type questions with six possible responses was unnecessary, time consuming, and no more informative than a simplified numerical response.

Participants were also asked about the feeding method (frequency of self-fed vs. spoon-fed) and, for the HV survey, this question was simplified. Twenty-one (17%) participants reported never offering eggs to their child. A question on eggs was, thus, added to the redraft of the questionnaire, in addition to meat and fish questions.

Commercial baby foods were moderately popular, with 57 (47%) participants offering these weekly or less, and 15 (14%) offering them daily. In order to assess their consumption accurately, this was included as an individual item on the redrafted questionnaire, with clarity as to their definition.

### 3.2. Stakeholder Consultation

There was substantial enthusiasm for routine questions on solid feeding from HV teams. Researchers, HV managers, and the professional nurse advisor agreed that there should be a manageable number of questions and that these should only include items that had the potential to identify important dietary issues. The responses needed to be suitable for inclusion within the electronic health record. Most data entered onto EMIS is recorded using READ codes, which is a complex and cumbersome clinical terminology system, that would require detailed encoding of every possible response. To avoid this, we decided instead to use numerical responses, mostly frequency counts, with two columns, to give parents and HV staff the opportunity to report weekly or daily frequency of consumption. The questions could not be added to the EMIS web for the purpose of the study. Therefore, an online survey platform was used to imitate the EMIS web as closely as possible.

### 3.3. Health Visiting Survey

#### 3.3.1. Survey Results

Two HV teams, covering seven primary care centers in NHS GGC, chosen for diversity in socioeconomic profiles and availability, took part in the pilot. They tested the questionnaire with parents/caregivers of the infants within their caseloads aged 12 months in December 2015 and January 2016. Questionnaires were completed for 187 (59%) of an eligible 319, but 170 (91%) of these were returned via the paper questionnaire.

These revealed that 75 (40.1%) infants had been breastfed at some point, but 38 (20.3%) infants were breastfed to six months, and 16 (8.6%) infants were breastfed for 12 months or longer. By the time of the questionnaire, only half were taking any formula milk and 62% were drinking unmodified cow’s milk. Sixty-four (32.2%) infants were regularly given sugar sweetened beverages, with 48 of those (26.8%) having these daily. The median age of first complementary foods was six months and 3% of infants were introduced to solid foods before four months. Two thirds of infants were never given recommended Healthy Start [25] or similar vitamin drops.

Meat was the most popular form of protein, but 10% never ate meat, while 38% did not eat eggs, and 20% did not eat fish. Nearly two-thirds were still eating some commercial baby foods. The three fruit and vegetable flavor categories assessed were each consumed at least once per week by more than 80%. Sugary snacks were given to 45 (24.1%) infants on a daily basis, with a further 103 (55.1%) having them every week or multiples times per week.

The top three foods or food categories reported as disliked by infants were eggs (11%), bitter vegetables (8%), and sweet vegetables (5%), but half of the infants were reported to have no disliked foods (54%). The great majority of infants (95%) were already feeding themselves finger foods, but 77 (41%) were still not using a spoon to feed themselves. Parents/caregivers found a question on how often disliked foods should be reoffered difficult to understand or answer. Since the question was phrased to assess parents’ knowledge of how many times they should offer foods, HV feedback suggested that this came across as a ‘test’ for parents, to which they were often unsure of the correct answer. This further augmented a lack of trust from parents/caregivers and an unwillingness to continue answering questions. Parents/caregivers for around half of the children reported signs of satiation (pushing food away/closing mouth/turning head) and dislike (holding food in mouth/spitting food/ throwing food) at least weekly, but crying during meals and gagging was seen rarely.

Many paper surveys were returned with both the ‘times per day’ and ‘times per week’ columns filled for all or some of the questions, but it was clear from the responses that frequencies per day were most useful for drinks, while frequencies per week were most suitable for foods and behaviors.

#### 3.3.2. Staff Interview Results

Lastly, qualitative feedback, obtained from eight HV team members reflecting on their experience of testing the questionnaire resulted in three consistent themes. These were (i) avoiding parent discomfort, (ii) practicality and clarity, and (iii) nutrition/feeding knowledge and capacity. Quotations to demonstrate those typical of participants can be seen in Table 5.

Avoiding parent discomfort: It was clear that rapport between staff and parents was an important element in successful data collection. Where parents were less trusting, there was some discomfort with data being recorded electronically regarding their practices. This was an important contributor to the use of paper questionnaires. HV staff also felt that some of the questions caused parents to feel as though they were being ‘tested’ for a right or wrong answer, particularly in the item assessing how often they re-offered refused foods. Similarly, HV staff felt that parents were sometimes reluctant to report certain behaviors, such as turning their head during feeding or crying during feeding, out of worry that this would come across as problematic. Some staff thought that parents were not likely to report feeding practices honestly, particularly if their practices were not in line with recommendations, and that parents reported what they felt staff wanted to hear.

One staff member suggested that allowing the parent to complete the questionnaire in private or in writing, by themselves, might tackle some of these barriers.

Practicality and clarity: Issues relating to practicality were important aspects of the feedback. HVs cited the ease of use for paper resources as opposed to the online software with the need for Wi-Fi access as an explanation for the high use of paper questionnaires. The choice of units given on the questionnaire (times per day or times per week) caused confusion and, on the paper version, staff did not always realize that responses needed to be numerical. Staff did not always have time to collect data particularly as the data collection took place over the Christmas period. However, others had been enthusiastic to carry out data collection, since they found that it echoed the nutrition conversation they would routinely have. Staff commented that it reminded them to discuss aspects of feeding and to ask about Healthy Start.

Nutrition/feeding knowledge and capacity: Most staff members felt that the questionnaire was exhaustive and covered everything they found to be important. Some expressed that the questionnaire allowed a better insight into what was going on than would otherwise have been possible, but others suggested that the questionnaire would not capture some common aspects of poor nutrition, such as high fat foods, very large portion sizes, or inappropriate foods/beverages (e.g., tea). Some parents were perceived to have insufficient capacity to follow dietary recommendations. Generally, staff felt they already knew enough to provide feeding advice, but others found the fact sheet helpful in backing up their advice.

## 4. Discussion

Scottish Government policy on maternal and infant nutrition states that, “the Scottish Government wants to ensure that all children have the best possible start to life, are ready to succeed and live longer, healthier lives” [26]. This work was carried out in the weeks following the introduction of electronic records among staff, and was, therefore, extremely timely in assessing the potential addition of this nutrition surveillance during a period of transition, and its acceptability among staff. In the interim, the system has been rolled out across NHS GGC, providing a single health record of all families. Feeding and diet are routinely discussed with families as infants progress to solid foods, and these conversations would, in the past, usually have been recorded in paper records. However, with the advent of electronic patient records (EPR), it is not clear how or where these should be recorded to enable consistent recording and importantly capturing quantitative data, which would assist in identifying nutritional issues that require further promotion among parents/caregivers. The revised Universal Health Visiting Pathway in Scotland has introduced a Child Health Review between 13 and 15 months, which could be used to record information on diet and eating systematically with the EPR. This would ensure consistent recording and act as a stimulus to HVs to ask about diet and feeding in a way that will facilitate healthy eating habits. Furthermore, with the increase in contacts within the universal HV pathway, is it acknowledged that the HV will have enhanced opportunities to build on and strengthen the therapeutic relationship with the parents/caregivers, which enables more meaningful and outcome-focused discussions that will include the importance of supporting families focusing on healthy eating in achieving this. Evidence underpinning the new pathway emphasizes the importance of connecting with families and providing “person-centeredness” [27,28]. No standard questions exist for assessing diet and feeding use beyond the early weeks. Therefore, this study tested different approaches to data gathering and refined the wording of questions to be suitable to allow maintenance of rapport with families.

In the online survey, our data showed infant feeding practices generally in line with recommendations. This is likely not representative given that we recruited from parenting sites and pages among parents who may be more likely to engage with guidelines and seek out information. We did, however, learn which items within the long lists of foods and eating behaviors could be logically combined for generating more useful data. This allowed us to assess fruit and vegetables consumption frequency and variety by narrowing the survey items to sweet starchy vegetables, sour fruit, and green bitter vegetables for the second survey. We also identified key foods that had been omitted, specifically sugar sweetened beverages and sugary snacks, and refined the wording of other questions. In the consultation, we learned about the constraints of the EPR and developed an approach, using only numerical answers to questions that made integration easier, but had the potential to increase confusion for staff completing it, at least initially. However, the preliminary data then allowed us to simplify these numerical responses. Additionally, the online preliminary survey allowed us to identify infant responses to feeding, which were inter-correlated, and we hypothesized their meaning that pushing food away, closing the mouth when offered food, and turning the head when offered food were likely to indicate fullness, while holding food in mouth, spitting food, and throwing food likely indicate dislike. Crying/screaming and gagging were the rarest behaviors and we theorized that these are likely to indicate more serious feeding issues. While these theories were not tested, we felt it important that the behaviors were still captured and, therefore, the grouped items were tested in the HV survey.

The pilot with HV teams tested the acceptability and feasibility for staff asking these questions. It also achieved a survey of parents, representative of HV caseloads, to establish which issues are common and relevant to parents. These data were less consistent with feeding recommendations, with low rates of vitamin supplementation (which is recommended in the UK), high intake of sugary drinks and snacks, and high rates of commercial baby food use. The latter is important since our previous work has shown that commercial baby foods most commonly consist of sweet-tasting vegetables and fruits, with significant contribution to sugar intake [29], which might influence development of sweet preferences in late childhood. The median age of complementary feeding was, however, closely in line with the recommended six months for the majority. HV feedback emphasized that they generally appreciated the scheduled window for nutrition conversations but were mindful of putting parents/caregivers at ease, and this must be built into any future routine survey in terms of language.

At the end of this process, we have been able to use the outputs of the preliminary questionnaires to eliminate and reword questions, which allowed us to develop a much shorter, more streamlined, definitive set of questions suitable for routine use at the new 13–15 months Child Health Review, and this is timely given the recent rollout of the revised pathway. Although this age window is toward the latter end of the complementary feeding period, it is the only one suited to the data collection given immunizations and other priorities during earlier visits, but could still allow measurement of outcomes for infant feeding interventions during earlier infancy. Assessing feeding at this age could still provide an opportune time to correct potentially harmful feeding behaviors within the first two years of life.

HVs made the research team aware of difficulties of electronic completion, which are likely to affect future routine use within EPR. The pilot was undertaken right at the start of EPR use in NHS GGC and it is to be expected that staff will be more comfortable now that it is better established. While some HVs seemed to also favor the paper approach for the dietary questions, this would reduce its usefulness for health promotion where individual items might trigger tailored advice.

The obvious limitation raised by HVs in their feedback is the time such questions take to ask and record. However, the survey responses made it possible to identify questions that could be combined or omitted altogether, because they were less clear or reflect rarer issues. The effect of this is a reduction from the complex multi-item online survey (supplementary material A) to two pages of 24 questions in the pilot (GI 1) down to just one side of 18 questions, which were recently confirmed and not presented in this paper, but are due to be incorporated to the local EPRs in May 2019.

This study had a number of strengths limitations. The preliminary online survey produced data that allowed the research team to reassess how useful survey items were and develop these further. However, it must be noted that this was among a convenience sample of parents who use social media, and is not necessarily representative. A strength of the second survey was that it included all children turning 12 months within three diverse districts of GGC and was likely to be more representative of the local population. For the HV qualitative data collection, interviews were not audio recorded and, therefore, not transcribed verbatim. Furthermore, the analysis was completed by only one researcher on a small number of interviews.

## 5. Conclusions

This study iteratively assessed survey items, which are both useful and acceptable to parents, staff, and researchers for routinely quantifying dietary diversity in infancy via health visiting teams and electronic health records. This information could be used to describe the nutritional strengths and vulnerabilities of individual areas and sub-populations and inform future health improvement activities in the future.

## Figures and Tables

**Table 1:** Study stages.

a.	An online survey of parents of infants aged 9–15 months, investigating milk and complementary feeding practices, the results of which we used to assess the use of individual survey items in generating valid and useful information related to adherence of formal recommendations.
b.	Development of a second more succinct and appropriate questionnaire using results from stage (a), in addition to stakeholder consultation, to be compatible with the electronic record system, which is used in the field by HV teams.
c.	Piloting of questionnaire by HV teams for infants ages 12 months, followed by semi-structured interviews to assess staff members’ experience of using the questionnaire, and their attitudes toward incorporating this into routine visits.

**Table 2:** Questionnaire used for the Health Visitor pilot with parents/caregivers of 12-month-old children.

Was your child breastfed? If so, until what age? If not at all, put 0.	Age: _________	
What age was your child when you first introduced solid foods?	Age: _________	
Does your child ever drink any of the following: If so, approximately how often? If not, put 0 times per week.	Insert number into either:	
	**Times per day or**	**Times per week**
Breast milk?	_________	_________
Formula milk?	_________	_________
Cow’s milk?	_________	_________
Diluting juice, fruit juice, fruit shoots, or fizzy drinks? (which are not sugar-free)	_________	_________
Any other kind of milk such as soya or goat’s milk? If so, what kind?	_________	_________

Does your child ever eat any of the following: If so, approximately how often?	**Times per day or**	**Times per week**
Eggs?	_________	_________
Meat? (Includes burgers, sausages, nuggets etc. but not commercial baby foods)	_________	_________
Fish?	_________	_________
Commercial baby foods? (including jars, baby biscuits, and savory snacks, pouches, ready meals, and any ready-made foods for babies)	_________	_________
Sweet starchy vegetables? i.e., sweet potato, carrot, parsnip, butternut squash (excluding in commercial baby food)?	_________	_________
Green leafy vegetables? i.e., cauliflower, cabbage, kale, broccoli, brussels sprouts, spinach or similar (excluding in commercial baby foods)	_________	_________
Does your child ever eat any of the following: If so, approximately how often?	**Times per day or**	**Times per week**
Any solid, sour (sharp), uncooked fruits such as orange, tangerine, lemon, green apple (excluding juice, smoothies, and commercial baby foods)?	_________	_________
Any sweet snacks, such as biscuits, cake, pastries, chocolate, or dried fruit?	_________	_________
Finger foods eaten by himself/herself?	_________	_________
Spooned foods eaten by himself/herself?	_________	_________
Healthy Start vitamin drops or similar supplements?		
Are there any foods, tastes, or textures your child really does not like?	Answer: _______	
On average, how many times would you try offering your child a food before deciding that they do not like it?	No. tries:_______	
Does your child ever do the following when offered food: If so, approximately how often?	**Times per day or**	**Times per week**
Push food away or close mouth or turn head?	_________	_________
Hold food in mouth or spit food or throw food?	_________	_________
Cry during meals?	_________	_________
Gag on food?	_________	_________
Did the parent/care-giver mention using ‘Baby-Led Weaning’ during the conversation? If yes, tick [ ]		

**Table 3:** Specific fruit and vegetables to which infants had been exposed in the online survey.

Vegetable	*n* (%) Exposed	Fruit	*n* (%) Exposed
Carrot	119 (97.5)	Banana	112 (91.8)
Broccoli	111 (90.9)	Strawberries	107 (87.7)
Peas	109 (89.3)	Apple	99 (81.2)
Sweet Potato	104 (85.3)	Mango	86 (70.5)
Cucumber	100 (81.9)	Blueberries	84 (68.9)
Tomato	100 (81.9)	Melon	81 (66.4)
Sweetcorn	99 (81.2)	Raspberries	78 (63.9)
Pepper	96 (78.7)	Orange	77 (63.1)
Cauliflower	94 (77.1)	Peach	67 (54.9)
Butternut Squash	88 (72.1)	Pineapple	60 (49.2)
Onion	83 (68.0)	Kiwi	55 (45.1)
Beans	69 (56.6)	Cherries	32 (26.2)
Turnip	52 (42.6)	-	-
Lettuce	49 (40.1)	-	-
Cabbage	40 (32.8)	-	-

**Table 4:** Correlations between infant reactions to food in online survey.

	Turn away	Push Food away	Close Mouth	Cry/Scream	Gag	Hold in Mouth	Spit out	Throw
Turn away	-	0.606 **	0.582 **	0.281 **	0.199 *	0.164	0.295 **	0.237 **
Push away		-	0.574 **	0.350 **	0.213 *	0.277 **	0.357 **	0.388 **
Close mouth			-	0.277 *	0.160	0.125	0.305 **	0.281 **
Cry/scream				-	0.236 **	0.226 *	0.233 *	0.257 **
Gag					-	0.278 **	0.056	0.129
Hold in mouth						-	0.405 **	0.185 *
Spit out							-	0.434 **
Throw								-

* *p* < 0.05; ** *p* < 0.05.

**Table 5:** Quotations from health visiting staff after the Pilot.

Theme	Typical Comment(s)
(i) Avoiding parent discomfort	*“As for using paper, I really didn’t have a choice. Using electronic questionnaires is just much too time consuming—the parents are used to seeing paper and if they see the iPad coming out it just makes everything more difficult.”*
(ii) Practicality and clarity	*“It was no problem. I would have been talking about milk and everything anyway. It was only five minutes extra after maybe 45 min of being there anyway.”* *“It’s mainly a connectivity issue, it takes forever to get connected, if you can at all.”*
(iii) Nutrition/feeding knowledge and capacity	*“Some parents would say the child was not a fussy eater, but then going through the questionnaire they will say “he does not eat that, he does not eat that”… and you will realize they are”.* *“I thought it was good, not confusing at all. It is good to explain that they need green veg and things too.”* *“Not really, maybe the issue of gagging. It came up a lot actually, when to add lumpy foods, as there was a lot of concern about gagging. I can imagine at 8 months a lot of them not having introduced lumpy foods even though they should have.”* *“I find that the parent’s response depends on their cognitive abilities. Some of them you will just zip through. They will know their child well and know the answers off the top of their head.”*

## Supplementary Materials

The following are available online at https://www.mdpi.com/1660-4601/16/10/1722/s1, Supplementary Material A: Diet and behaviour questions: online survey; Supplementary Material B: Fact sheet for health visitor use.
