# Supplementary material for: Assessing the Potential for Integrating Routine Data Collection on Complementary Feeding to Child Health Visits: A Mixed-Methods Study
Source: Int J Environ Res Public Health. 2019 May 16;16(10):1722. doi: 10.3390/ijerph16101722 (PMC6571620; doi:10.3390/ijerph16101722)
Supplement: Supplementary file 1 [file ijerph-16-01722-s001.zip › supplementary material A.docx]

Diet and behaviour questions: online survey

| At what age did you feed your child his/her first solid food? | | ______ months | | | | | | | | | | | | | | | |
| --- | --- | --- | --- | --- | --- | --- | --- | --- | --- | --- | --- | --- | --- | --- | --- | --- | --- |
| Was your baby ever breastfed? | | No, not at all | | | | Yes, until the age of _____ months | | | | | | | Yes, I’m still breast feeding | | | | |
|  | | [ ] | | | | [ ] | | | | | | | [ ] | | | | |
| How often does your child eat  (please tick one answer for each): | | Never/ rarely | | | At least once a month but not weekly | | | | At least once a week but not daily | | | | | Once daily | | | More than once daily |
| Meat/fish/chicken/poultry | | [ ] | | | [ ] | | | | [ ] | | | | | [ ] | | | [ ] |
| Eggs | | [ ] | | | [ ] | | | | [ ] | | | | | [ ] | | | [ ] |
| Breast milk | | [ ] | | | [ ] | | | | [ ] | | | | | [ ] | | | [ ] |
| Formula milk | | [ ] | | | [ ] | | | | [ ] | | | | | [ ] | | | [ ] |
| Whole (doorstep) milk | | [ ] | | | [ ] | | | | [ ] | | | | | [ ] | | | [ ] |
| Any fruit (not juice) | | [ ] | | | [ ] | | | | [ ] | | | | | [ ] | | | [ ] |
| Any vegetables (not including soup, sauces) | | [ ] | | | [ ] | | | | [ ] | | | | | [ ] | | | [ ] |
| Do you give your child any multivitamin drops? | | Daily | | | Most days | | | | Some days | | | | | Never | | |  |
|  |  | [ ] | | | [ ] | | | | [ ] | | | | | [ ] | | |  |
| If so, what sort do you give? | | Healthy Start | | | Other over the counter brand | | | | | | Other prescribed brand | | | | | | |
|  | | [ ] | | | [ ] | | | | | | [ ] | | | | | | |
| Which fruit and vegetables does your child most like to eat? | |  | | | | | | | | | | | | | | | |
| Which fruits in the list below has your child ever tried? (tick all that apply) | | | | | | | | | | | | | | | | | |
| [ ] Apple | [ ] Kiwi fruit | | | | | | | [ ] Peach / nectarine | | | | | | | | | |
| [ ] Banana | [ ] Mango | | | | | | | [ ] Pineapple | | | | | | | | | |
| [ ] Bluberries | [ ] Melon | | | | | | | [ ] Raspberries | | | | | | | | | |
| [ ] Cherries | [ ] Orange / tangerine / satsuma | | | | | | | [ ] Strawberries | | | | | | | | | |
| Which of the vegetables in the list below has your child ever tried? (tick all that apply) | | | | | | | | | | | | | | | | | |
| [ ] Baked beans | [ ] Cauliflower | | | | | | | [ ] Red or green pepper | | | | | | | | | |
| [ ] Broccoli | [ ] Cucumber | | | | | | | [ ] Sweetcorn | | | | | | | | | |
| [ ] Butternut squash | [ ] Lettuce | | | | | | | [ ] Sweet potato | | | | | | | | | |
| [ ] Cabbage | [ ] Onions | | | | | | | [ ] Tomatoes | | | | | | | | | |
| [ ] Carrots | [ ] Peas | | | | | | | [ ] Turnip | | | | | | | | | |
| Are there any other fruits or vegetables your child has tried? |  | | | | | | | | | | | | | | | | |
| Now some questions about how you child eats. Please tick one answer for each | **Entirely self feeds** | | **Mostly self feeds** | | | **Half and half** | | **Carer mostly feeds** | | | | **Carer always feeds** | | | | **Not given** | |
| Who feeds the child foods that are served on a plate and eaten with a spoon or fork? | [ ] | | [ ] | | | [ ] | | [ ] | | | | [ ] | | | | [ ] | |
| Who feeds the child finger foods, pieces of fruit, biscuits)? | [ ] | | [ ] | | | [ ] | | [ ] | | | | [ ] | | | | [ ] | |
| **How often does your child do the following when offered food:**  (please tick one answer for each) | **All the time** | | | **Most of the time** | | | **Sometimes** | | | **Rarely** | | | | | **Not at all** | | |
| Turns away | [ ] | | | [ ] | | | [ ] | | | [ ] | | | | | [ ] | | |
| Pushes food away | [ ] | | | [ ] | | | [ ] | | | [ ] | | | | | [ ] | | |
| Closes mouth when offered food | [ ] | | | [ ] | | | [ ] | | | [ ] | | | | | [ ] | | |
| Cries/ screams | [ ] | | | [ ] | | | [ ] | | | [ ] | | | | | [ ] | | |
| Gags on food | [ ] | | | [ ] | | | [ ] | | | [ ] | | | | | [ ] | | |
| Holds food in mouth | [ ] | | | [ ] | | | [ ] | | | [ ] | | | | | [ ] | | |
| Spits out food | [ ] | | | [ ] | | | [ ] | | | [ ] | | | | | [ ] | | |
| Throws food | [ ] | | | [ ] | | | [ ] | | | [ ] | | | | | [ ] | | |
