# Supplementary figures and images for: Assessing the Potential for Integrating Routine Data Collection on Complementary Feeding to Child Health Visits: A Mixed-Methods Study
Source: Int J Environ Res Public Health. 2019 May 16;16(10):1722. doi: 10.3390/ijerph16101722 (PMC6571620; doi:10.3390/ijerph16101722)

**Fact Sheet for HV Use**
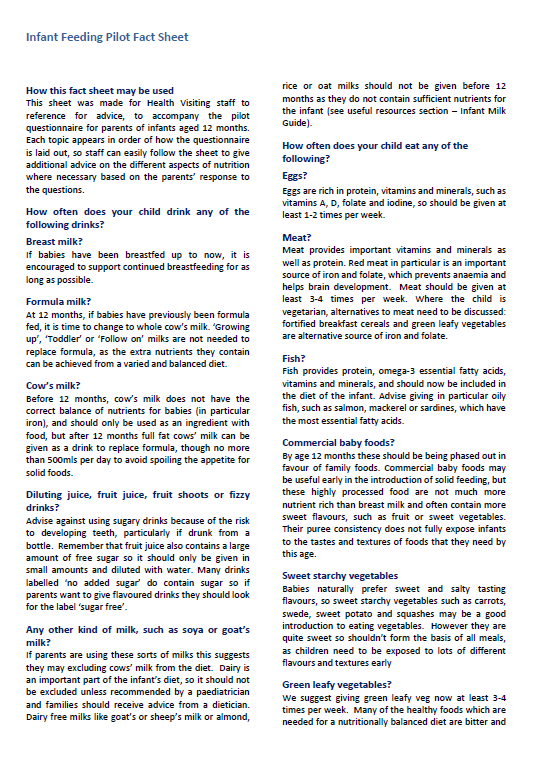


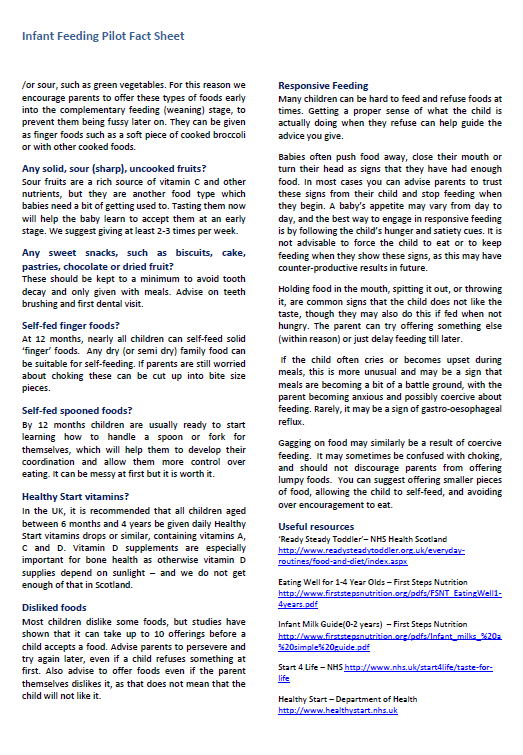

Supplement: Supplementary file 1 [file ijerph-16-01722-s001.zip › supplementary material B.docx]
